# Development and validation of new predictive equations for resting energy expenditure in physically active boys

**DOI:** 10.1038/s41598-023-31661-1

**Published:** 2023-03-20

**Authors:** Edyta Łuszczki, Paweł Jagielski, Anna Bartosiewicz, Katarzyna Dereń, Piotr Matłosz, Maciej Kuchciak, Łukasz Oleksy, Artur Stolarczyk, Artur Mazur

**Affiliations:** 1 Institute of Health Sciences, Medical College of Rzeszów University, Rzeszow, Poland; 2 Department of Nutrition and Drug Research, Institute of Public Health, Faculty of Health Sciences, Jagiellonian University Medical College, Krakow, Poland; 3 Institute of Physical Culture Sciences, Medical College of Rzeszów University, Rzeszow, Poland; 4 Faculty of Health Sciences, Department of Physiotherapy, Jagiellonian University Medical College, Kraków, Poland; 5 Orthopedic and Rehabilitation Department, Medical University of Warsaw, Warsaw, Poland; 6 Institute of Medical Sciences, Medical College of Rzeszow University, Rzeszow, Poland

**Keywords:** Nutrition, Epidemiology

## Abstract

Measurement or estimation of resting energy expenditure (REE) should be the first step in determining energy demand in physically active boys. The purpose of this study was to develop and validate new equations for resting energy expenditure in male children and adolescents practicing soccer. The cross-sectional studywas carried out among 184 boys in the derivation group and 148 boys in the validation group (mean age 13.20 ± 2.16 years and 13.24 ± 1.75 years, respectively). The calorimeter and device for assessing body composition by bioelectrical impedance analysis (BIA) were used. Model of multiple regression showed that REE can be predicted in this population with Eq. (1) (with height and weight data) or Eq. (2) (with age, height, and fat free mass data). Predictive Eq. (1) had an average error of 51 ± 199 kcal and predictive Eq. (2) − 39 ± 193 kcal. Cohen's d coefficient was 0.2, which confirms the small difference. The bias was 4.7% and 3.9%, respectively. The accuracy was 61.2% in the population for predictive Eq. (1) and 66.2% for predictive Eq. (2). Therefore, the new equations developed and validated in this study are recommended for the estimation of REE in physically active boys, when the use of IC is not feasible or available.

## Introduction

The energy expenditure values among young people were developed in 2008, and the compilation provides values of metabolic equivalents (MET) for each type of activity, averaging them for age, sex, and other characteristics^1^. In some cases, adult values have been considered^2^. Nevertheless, children's physical and mental characteristics vary compared to adults. Physical activity (PA) is crucial for both the health and correct development of children and adolescents. Therefore, from a public health perspective, increasing PA is one of the most important ways to improve overall health^3^.

The proportions of the body compartments: fat mass (FM) and fat free mass (FFM) change during growth and development. Consequently, these components can be determinant when analyzing the physical fitness of children and adolescents^4^. In this context, physical fitness has been documented as a key determinant of a healthy lifestyle^5^. Research shows that of the top ten extracurricular sports among children and adolescents, the most popular is soccer. It is followed by swimming, running, and cycling^6^, and boys and girls are very different in this respect.

In Poland and Europe, the popularity of sports schools for the young generation is growing. Students in sports championship schools attend at least 16 h of sports activities per week. Many sports championship schools, which educate in team disciplines, such as soccer or volleyball, field teams of outstanding students in league competitions. Appropriate energy intake in the diet is a key for the population of young athletes. Most of these children eat at school, some live in a boarding school, and eat all their meals there. The measurement or estimation of resting energy expenditure (REE) should be the first step in determining the energy demand of these children and adolescents^7^. REE constitutes 60–70% of the total energy requirement for most people^8^. However, the REE level should be set accordingly as a valuable tool in the development of food rations, the menu in schools, and nutrition plans. It improves athletic performance and prevents weight loss in children and adolescents.

Energy balance is integral for boys to sustain optimal growth and development, with additional nutritional intake required to offset the increased energy cost of training. Studies have investigated the nutritional intake in adult professional soccer players^9,10^. However, a relatively limited number of studies have investigated the nutritional intake of children and adolescents soccer players^11^. The findings of these studies have presented suboptimal energy intake relative to estimates of energy expenditure^11^. Therefore, correctly indicating the REE and then increasing it by the expenditure of physical activity will correctly estimate the total daily energy demand.

Despite REE can be calculated very precisely using laboratory methods such as indirect calorimetry (IC), for children and adolescents in sports predictive equations from the literature are also used. There are many equations created for children in the literature, however, these were not formulated for physically active boys or young athletes. In addition, the method for evaluating REE is time-consuming, cost-prohibited, and requires sophisticated tools; therefore, sports trainers, teachers, or people related to sports use the predictive equations developed. The creation of new predictive equations will be very useful for small sports clubs where, due to lack of funds and equipment, the use of IC is not possible. It is widely known that the accuracy of REE predictive equations is characteristic of the population for which they were formulated, and should not be used for groups different from what was originally intended^12^. The results of the literature agree that REE is elevated with excess weight^13,14^. Observation presents the FFM as the strongest indicator that affects the REE^15^. FM is also a component of body composition, which could affect REE, but the research is inconclusive^16,17^.

In 2020, we conducted a cross-sectional study that compares the precision of REE known from previous publications in the literature with the values derived from IC measurement among boys who play soccer. Our results showed that most ready-made equations underestimate REE, which can be a problem, especially if we define the total energy demand of children, who, due to intensive physiological development and regular training, require more calories per day. In conclusion, to date, the best predictive equation has not been created for boys activity undertaking intense physical activity^18^. As a result of the still low availability and high cost of IC devices, further research was needed that could allow the formation of a special equations for physically active boys. Therefore, the objective of this study was to validate the new predictive equations for active male children and adolescents training soccer.

## Methods

### Subjects and new predictive equations

In 2020 we conducted a coss-sectional study among 184 boys aged 10 to 16 using a calorimeter and a device for assessing body composition by means of electrical bioimpedance using a segment analyzer. A detailed description of the group and the methodology has already been published^18^.

In September 2021 we conducted a a cross-sectional validation study among 148 boys aged 10–16 years. With the start of school classes without restrictions and online classes, invitations were sent to all Sports Championship School directors (13 schools) in the Podkarpackie Voivodeship (south-eastern Poland). Of the six schools that agreed, an invitation was sent to all parents or guardians of boys attending these schools. However, due to the ongoing pandemic in Poland, we obtained fewer research approvals than in previous years (before the pandemic). Of the six selected schools, 183 parents agreed to have their children examined. Of these, 35 children did not complete the study due to injury or discontinued the study for any reason (e.g. illness). The inclusion criteria were as follows: male, playing soccer, age between 9 and 16, training for minimum 2 years, training 3 times a day/match once a week, and with the consent of parents/guardians to participate in the study. The exclusion criteria were: female, age < 9 and > 16 years, training < 2 years, training < 3 times a day, a functional state that does not allow for self-maintenance of a standing position, illness or injury, a lack of desire to participate in the study or strong pre-test anxiety, and being absent from school on assessment days. 183 parents/guardians agreed to participate in the study. Of these, 35 children did not complete the study due to injury or discontinued the study for any reason. All subjects were healthy. In the last 6 months, no weight loss or infection with increased fever were reported. They did not use any drugs. Parents/guardians and participants gave their informed consent to participate in the study.

### Assessments

All examinations were conducted in the laboratories of Rzeszow University by experienced researchers between January and May 2022. Participants came forward to the laboratory from 7:00 to 10:00 a.m. at controlled temperature. The temperature at the location of the measurements was controlled (22–25 °C).

### Anthropometric measurements, body composition

Before taking the measurements, the participants received precise information about the course of the study. To minimize the risk of bias in body composition analysis, the bladder was emptied. Height measurement was made with a height meter (Seca 213) with an accuracy of 0.5 cm. The boys took off their shoes and stood with their backs to the stadiometer in an upright position. The average of three measurements was used for analysis.

Body composition was measured using bioelectrical impedance analysis (BIA, 6.25 kHz/50 kHz, 90 µA). The TANITA MC-980 MA (Tanita, Tokyo, Japan) was used. The analyzer is equipped with 8 electrodes, of which 4 are built into the platform, while the others are placed in the handles. Participants were asked to remove footwear and socks. Measurements were made in underwear, standing in designated places on the platform. According to the Tanita MC-980 PLUS MA manual, accurate measurement requires setting up the machine as level as possible. The adjustable feet were rotated in 4 positions so that the bubble of the level indicator was in the middle. Participants stood upright on the platform with their legs extended, placing their feet so that they touched the front and rear electrodes, ensuring that the weight was evenly distributed on both feet. The person examined held handles in their hands that were taken from the body at an angle of 35–40.

### Resting energy expenditure

Resting energy expenditure (kcal/day) was measured using a Cosmed Quark RMR indirect calorimeter (Rome, Italy) with a ventilated canopy hood and a disposable antibacterial filter. Full service of all measurement instruments was performed prior to the study, and daily calibration was performed according to the manufacturer's instructions. The evidence-based protocol for measurement of resting energy expenditure by IC was adopted in the study and clearly discussed (Table 1).

**Table 1:** Evidence-based guidelines for measurement of resting metabolic rate with IC.

Criteria	Guidelines for measurement	Study group recommendation
Fasting (thermic effect of food)	Minimum fast 5 h after meals or snacks (Grade II), 4 h after small meal if longer fast is clinically inappropriate (Grade II)	All recommendations concerning preparations for the study were outlined, including: having rest minimum for 20 min, abstention from nicotine for minimum 2 h, refraining from the consumption of meals 12 h before the test, refraining from drinking beverages with caffeine and alcohol content for the last 48 h before the test, as well as refraining from participation in a physical activity for the previous 14 h The method of conducting the study was explained in detail and each study participant had the opportunity to visit the test rooms beforehand and familiarize themselves with the equipment so that it did not raise concerns or cause anxiety in the researched group
Alcohol ingestion	Minimum abstention from alcohol for 2 h (Grade III)	
Nicotine ingestion	Minimum abstention from nicotine for 2 h (Grade II)	
Caffeine ingestion	Minimum abstention from caffeine for 4 h (Grade II)	
Rest periods	Rest 10–20 min (Grade III)	
Physical activity restriction	Minimum abstention from moderate aerobic or anaerobic exercise for 2 h before test (Grade II), for vigorous resistance exercise abstention of at least 14 h (Grade III)	
Environmental conditions	Allow a room temperature of 20–25 °C (68–77°F) (Grade III) Ensure each individual is physically comfortable with measurement position during the test and repeated measures are in the same reclined position (Grade V)	The rooms had a controlled temperature between 22 and 25 °C In addition, each participant had the opportunity to acclimatize in the environment by lying flat for 30 min
Gas collection devices	Use rigorous adherence to prevent air leaks (Grade III) Further studies comparing modern gas collection devices are needed in healthy and clinical populations (Grade V)	With these devices, exhaled gas was captured by a canopy (ventilated hood system) or a face mask connected to oxygen and carbon dioxide analyzers mounted on a metabolic cart. This is essential for correct measurement
Steady-state conditions and measurement interval	Discard initial 5 min. Then achieve a 5-min period with 10% CV^b^ for VO_2_^c^ and VCO_2_^d^ (Grade II)	We use a 20-min protocol in which the first 5 min of data are discarded and the remaining 15 min of data have a coefficient of variation of no more than 10%
No. of measures/24 h	Achieve steady state and one measure is adequate; if not, two to three nonconsecutive measures improve accuracy (Grade II)	1 measure/24 h
Repeated measures (daily to monthly variation)	Repeated measures vary 3%-5% over 24 h (Grade II) and vary up to 10% over weeks to months (Grade II)	–
Respiratory quotient (RQ)	RQ measures 0.70 or 1 suggest protocol violations or inaccurate gas measurement (Grade II)	The Quark RMR is a state-of-the-art metabolic system designed for accurate measurement of Resting Energy Expenditure (REE) and respiratory ratio (R), in a non-invasive way, through the measurement of oxygen consumption (VO2) and carbon dioxide production (VCO2) together with other ventilatory parameters. RQ was between 0.7 and 1.0

^a^Grade I—strong, consistent evidence; Grade II—somewhat weaker evidence and disagreement among authors may exist; Grade III—limited design quality; Grade IV—professional opinion only, no clinical trials; Grade V—no available studies.

^b^CV—coefficient of variation (standard deviation [mean of individual replicate measures] × 100).

^c^VO_2_—oxygen consumption.

^d^VCO_2_—carbon dioxide production.

The following statistical methods were used.

Descriptive statistics are presented in Table 2 (number (n), Me—median and standard deviation (SD)). Shapiro–Wilk test, allowed to test the compliance of the tested variable with the normal distribution and the Student's t-test or Mann–Whitney *U* test was used to check the differences between both groups for the analyzed quantitative or ordinal variables. The bias was calculated as the mean difference between the predicted value and the measured REE.

**Table 2:** Anthropometric characteristics of the study participants.

Variables	Derivation group (2020 year) N = 184					Validation group (2022 year) N = 148					
	Mean	SD	Me	Min	Max	Mean	SD	Me	Min	Max	*p*
Age [years]	13.20	2.16	13.00	10.00	16.00	13.24	1.75	13.24	10.01	16.43	0.8289
Height [cm]	162.91	14.90	166.00	132.00	191.00	161.01	13.51	160.50	128.00	193.00	0.2310
Weight [kg]	52.37	14.44	53.85	24.80	102.20	50.84	13.38	49.65	26.92	87.88	0.3233
BMI [kg/m^2^]	19.27	2.57	19.05	13.60	28.00	19.23	2.42	19.11	13.79	26.53	0.8259
Fat %	17.58	3.76	16.90	8.80	34.00	18.38	4.03	17.68	10.40	40.80	0.0597
FFM [kg]	43.17	12.02	44.40	20.60	79.60	41.52	11.21	39.70	22.70	72.40	0.2017

*Me* median, *SD* standard deviation, *p* t Student test, *BMI* body mass index, *FFM* fat free mass.

The agreement between measured and estimated REE values was evaluated by determining the bias in absolute values and as a percentage of the measured value and the corresponding limit of agreement (upper limit of agreement (ULA) = bias + 1.96 × SD; lower limit of agreement (LLA) = bias − 1.96 × SD). Additionally, Pearson’s product-moment correlation coefficient (r) and the coefficient of determination (R2) were calculated. The concordance correlation coefficient (CCC) as a measure of agreement was also determined.

The percentage of participants whose predicted REE value was within ± 10% of the measured REE was used as a precision measure. The heteroscedasticity was tested using the Bland–Altman method. The plots presented the difference between predicted and measured REE versus the mean of predicted and measured REE.

The PS IMAGO PRO 8.0 software (IBM SPSS STA-TISTICS 28) and the MedCalc software were used.

The adopted level of statistical significance was p < 0.05.

### Ethical consent

This research project obtains acceptance of Institutional Bioethics Committee at the University of Rzeszów (Resolution No. 2/01/2019). Parents/guardians and participants gave their informed consent to participate in the study.

## Results

### Characteristics of the study group

184 boys aged 10–16 years partake in the study when the new predictive equations were developed and 148 subjects aged 10–16 years in 2022 to validate the new equations. The descriptive characteristics of the study group are presented in Table 2.

### The findings

Model of multiple regression showed that REE can be predicted in this population with Eqs. (1) or (2), as follows:

Predictive Eq. (1):

1 $${\text{REE }}\left( {{\text{kcal}}/{\text{d}}} \right) \, = \, - {196}.{49 } + { 9}.{25 }*{\text{ Height }}\left( {{\text{cm}}} \right) \, + { 1}0.{2}0 \, *{\text{ Weight }}\left( {{\text{kg}}} \right)$$

(R = 0.84, p < 0.001; bias = 0, p = 0.93).

Predictive Eq. (2):

2 $${\text{REE }}\left( {{\text{kcal}}/{\text{d}}} \right) \, = { 359}.{45 }{-}{ 23}.{69}*{\text{ Age }}\left( {{\text{years}}} \right) \, + { 5}.{64 }*{\text{ Height }}\left( {{\text{cm}}} \right) \, + { 2}0.{36 }*{\text{ FFM }}\left( {{\text{kg}}} \right)*$$

(R = 0.86, p < 0.001; bias = 0, p = 0.9992).

For the first, the following data were used: height and weight (basic anthropometric data), and for the second, age, height, and fat free mass (body mass composition data). The R correlation coefficient indicates that there is a strong correlation between the results obtained from the developed predictive equation and the results measured by the IC method.

REE calculated by IC in the first group averaged 1844 ± 328 kcal and in the second group 1760 ± 357 kcal.

The validation group consisted of 148 boys. The results showed that both Eqs. (1) and (2) had a very low error in relation to the REE measured with the IC. Predictive Eq. (1) had an average error of 51 ± 199 kcal and predictive Eq.  (2) − 39 ± 193 kcal. Cohen's d coefficient was 0.2, which confirms the small difference. The bias was 4.7% and 3.9%, respectively. These values are a limit value that does not exceed 10% of the error; therefore, predictive Eq. (1) and (2) seem to be appropriate to be used in young sports people. The accuracy of the prediction was on a very high level in both two equations. The accuracy was 61.2% in the population for predictive Eq. (1) and 66.2% for predictive Eq. (2). This means that both equations are consistent (with an error of ± 10%) for more than 60% of the population. Accuracy of the predictive models is critical as it determines the quality of their predictions that form the scientific evidence. In our study 61.2% and 66.2% represent a high accuracy (Table 3).

**Table 3:** Validity of the resting energy expenditure.

	REE (kcal/d)		T-test	Bias kcal/d		LLA kcal/d	ULA kcal/d	Bias (%)		r	p-value	R^2^	p-value (linear regression)	CCC	Prediction		
	Mean	SD	p-value	Mean	SD			Mean	SD		(correlation)				Accurate %	Under %	Over %
REE derivation group	1844	328													100		
Equation (1)	1844	279	0.9345	0	172	− 337	337	0.8	9.0	0.84	< 0.0001	0.73	< 0.0001	0.84	73.4	10.9	15.7
Equation (2)	1844	167	0.9992	0	167	− 327	327	0.8	9.1	0.86	< 0.0001	0.74	< 0.0001	0.85	76.0	10.4	13.7
REE validation group	1760	357													100		
Equation (1)	1811	256	0.0022*	51	199	− 441	339	4.7	12.5	0.84	< 0.0001	0.70	< 0.0001	0.78	61.2	8.8	29.9
Equation (2)	1799	266	0.0154**	39	193	− 417	339	3.9	12.1	0.85	< 0.0001	0.72	< 0.0001	0.81	66.2	8.8	25.0

*REE* measured resting metabolic rate by IC, *SD* standard deviation, *Bias* difference between the predicted value and the measured REE, *LLA* lower limit of agreement, *ULA* upper limit of agreement, *bias%* bias in %, *CCC* concordance correlation coefficient, *Accurate %* percentage of subjects in which the error of the predictive equation was within 10% of the measured value, *Under %* percentage of subjects underestimated by the predictive equation with an error > 10% of the measured value, *Over %* percentage of subjects overestimated by the predictive equation with an error > 10% of the measured value.

*Cohen's d = 0.26.

**Cohen's d = 0.20.

**Figure 1:**
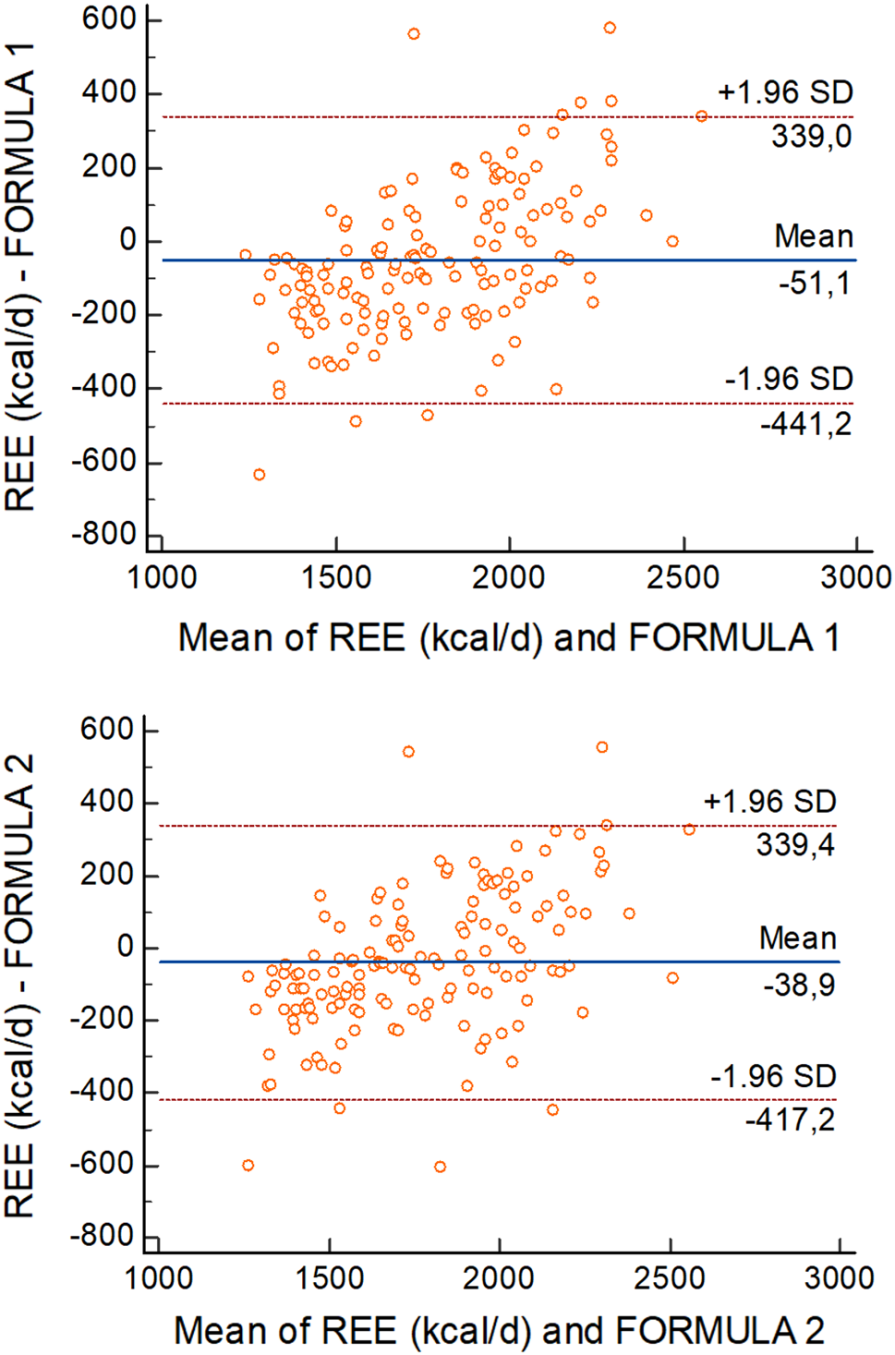
Agreement between measured and predicted resting energy expenditure (REE) using the Eqs. (1) and (2)—Bland Altman plot.

The mean value of the differences between measured and predicted REE (bias) is indicated by the solid line in the Fig. 1. The dashed lines delimit the 95% confidence interval. All regression lines were statistically significant at p < 0.0001, indicating a systematic bias.

## Discussion

In the present study, two new specific equations were developed and validated to predict REE in physically active male children and adolescents. A correct estimate of resting energy expenditure is essential for nutritional management and calculates total energy. When IC measurement is not feasible or available, ready equations became a valuable tool for estimating REE. However, the optimal predictive accuracy of these equations is obtained when used in subjects with the same characteristics as those in whom the equations were developed^19^.

To our knowledge, this is the first study available in the literature on the development and validation of new predictive equations to estimate REE in healthy boys who practice sports regularly. This is a very important issue because optimizing energy consumption is of fundamental importance in the population of young athletes for whom, in addition to energy expenditure associated with the development of the body, the energy demand due to intense physical activity also increases.

The mean REE value for physically active boys was 1844 kcal/day in the derivation groupand 1760 kcal in the validation group. It seems to be representative of other active male children and adults examined so far. The literature presented that among active men, REE values were 1858 kcal/day, 1788 kcal/day, 1808 kcal/day^20–22^ and 1834 kcal/day among 10 soccer players^23^. In one study investigating the dietary and activity regimes of adolescent soccer players in the UK, the mean daily energy deficit of − 3299 ± 729 kJ was observed^24^. The researchers noticed that the comparison of ready-to-use formulas to calculate REE in physically active people with IC shows that several of these equations underestimate or overestimate REE by up to 300 kcal^25^. The literature shows that this bias may be caused by differences in the metabolic activity of FM and FFM because most of these predictive equations are not based on body composition, but on total body weight^26^. The literature indicates that formulas having the smallest error deviations in the population of children and adolescents are the formulas of Schofield and FAO/WHO/UNU based on body weight and height. Rodriguez et al. observed that the FAO/WHO/UNU and Schofield formulas were suitable for predicting REE in children and adolescents^27^. Hofsteenge et al. showed that it was not a proper equation for obese adolescents and presented overestimation with a deviation of + 10.7% and mean squared prediction error 276 kcal/day^28^. On the other hand, a meta-analysis from 2020 presented that the Mifflin equation shows the highest precision in the 11–18 years old obese children and adolescents^29^.

According to our previous study, we found that the predictive equation for boys who are physically active regularly has not been developed to date. Although the use of the Institute of Medicine of the National Academies (IMNA) predictive equation gave the smallest error in the REE estimates, this equation and all the predictive equations used in these studies underestimated the REE of children and adolescents. The mean error ranged from 477 kcal/day for the Maffeis predictive equation to 182 kcal/day for the IMNA predictive equation^18^. Similar results were found in studies checking the accuracy of REE equations in sporting populations, especially in endurance sports^25,30,31^. De Lorenzo et al.^30^ found that the Cunningham equation overestimates in about 59 kcal/day measured REE. Harris Benedict and Mifflin formulas underestimated REE in 51 male athletes in various types of sports. Thompson et al.^25^ noticed that all mean predicted REE values were lower than those measured with IC, except for the Cunningham formula in male and female endurance athletes^31^. Due to the still low availability of IC devices, we decided that further research is needed that will allow us to generate special equations for physically active boys.

In our study, we identified appropriate predictive equations for the population of physically active boys who play male soccer. A linear regression analysis was performed to obtain two new predictive equations for REE in the study group. It is well known that predictive equations cannot replace REE measurements by IC, but the large, homogeneous group in our study allows us to conclude that these predictive equations can be used among young male soccer players. The results of the study can be applied with caution to similar groups of the population, taking into account the limitations of this study and the factors that affect the REE of athletes.

The first prediction equation, based on anthropometric parameters (body weight and height), is easier to use by dietitians and physicians during medical examination because it only needs a typical scale and growth meter/stadiometer. The second equation, based on body composition (FFM), age and height, is more population-specific than the predictive Eq. (1) because in most studies FFM was the main significant determinant of REE in the population^32–34^. However, it involves specific equipment and more time to measure body composition.

Nevertheless, in a similar random sample of 148 boys (validation group), both new equations predicted REE with a bias of 4.7% and 3.9% and were accurate for 61.2% [Eq. (1)] and 66.2% of the population. Furthermore, when an external validation was performed in an independent group of physically active boys, the REE estimated by both equations was significantly different from the measured REE, but the prediction was very high and similar to different studies that validated new predictive equations^35^. In the validation cohort, the two new equations present the same small mean difference and a similar SD of the differences between measured and predictive REE, although FFM has been shown to be the best predictor of REE in many studies.

In the literature, it is observed that FFM explains REE better compared to body weight. Differences in REE are known to be related to FFM, but genetics could also explain the difference in REE between populations. Therefore, the predictive equations for REE based on body composition are generally population-specific and therefore should be more appropriate^36^. Together with age and gender, physical activity has a significant influence on FFM. The issue of body composition and the influences of physical activity is quite different from REE. However, there is limited evidence on the evaluation of body composition and its dynamic changes in children and adolescents who train sports regularly^37^.

### Strengths and limitations

The study was the first known to develop and validate REE in physically active boys and represents two population groups, the derivation group (for which two prediction equations are developed) and the validation group. This is the first known study to create new equations to estimate REE in boys who play soccer regularly. Groups 1 and 2 were relatively large and representative.

The results should be considered to estimate REE only in a specific population of soccer, boys, Caucasian race, and at a specific age of 10–16 years. The group consisted of young boys who played sport regularly; therefore, our results could be confounded by overweight, obesity, and unintentional weight gain, therefore, our findings could not be generalized. The age range of the subjects in the experiment is from 10.01 to 16.43 years old, and their hydration status are always changing with their maturations, and it could influence on fat free mass measured by BIA.

## Conclusions

Our study allowed us to collect data to develop new predictive equations for male children and adolescents who play soccer. This is very important because REE is an essential element in evaluating and determining the diet of boys with high physical activity in the context of balancing their daily energy expenditure. It affects not only the maintenance of normal body weight composition and is related to the proper development and maintenance of health, but also the maintenance of optimal physical fitness in boys. Most ready-made predictive equations underestimate REE, which can be a problem, especially if we define the total energy demand of children, who due to intensive physiological development and regular training, require more calories per day. Therefore, the new predictive equations could be more appropriate for predicting REE in this population. These predictive equations can be very useful for small sports clubs where, due to lack of funds and equipment, the use of IC is not possible.

## Data Availability

The data presented in this study are not publicly available due to confidentiality reasons. These data are available on request from the corresponding author.
